# Targeting the gut microbiota with dietary fibers: a novel approach to prevent the development cardiovascular complications linked to systemic lupus erythematosus in a preclinical study

**DOI:** 10.1080/19490976.2023.2247053

**Published:** 2023-08-24

**Authors:** Javier Moleón, Cristina González-Correa, Iñaki Robles-Vera, Sofía Miñano, Néstor de la Visitación, Antonio Manuel Barranco, Natividad Martín-Morales, Francisco O’Valle, Laura Mayo-Martínez, Antonia García, Marta Toral, Rosario Jiménez, Miguel Romero, Juan Duarte

**Affiliations:** a Department of Pharmacology, School of Pharmacy and Center for Biomedical Research (CIBM), University of Granada, Granada, Spain; b Instituto de Investigación Biosanitaria de Granada, ibs.GRANADA, Granada, Spain; c Centro Nacional de Investigaciones Cardiovasculares (CNIC), Madrid, Spain; d Division of Clinical Pharmacology, Department of Medicine, Vanderbilt University Medical Center, Nashville, Tennessee, USA; e Department of Pathology, School of Medicine, Instituto de Biopatología y Medicina Regenerativa (IBIMER) University of Granada, Granada, Spain; f Ciber de Enfermedades Cardiovasculares (CIBERCV), Madrid, Spain; g Centre for Metabolomics and Bioanalysis (CEMBIO), Faculty of Pharmacy, Universidad San Pablo CEU, CEU Universities. Campus Monteprincipe, Boadilla del Monte, San Pablo, Spain

**Keywords:** Hypertension, endothelial dysfunction, fibers, gut dysbiosis, immune system, systemic lupus erythematosus

## Abstract

This study is to investigate whether dietary fiber intake prevents vascular and renal damage in a genetic mouse model of systemic lupus erythematosus (SLE), and the contribution of gut microbiota in the protective effects. Female NZBWF1 (SLE) mice were treated with resistant-starch (RS) or inulin-type fructans (ITF). In addition, inoculation of fecal microbiota from these experimental groups to recipient normotensive female C57Bl/6J germ-free (GF) mice was performed. Both fiber treatments, especially RS, prevented the development of hypertension, renal injury, improved the aortic relaxation induced by acetylcholine, and the vascular oxidative stress. RS and ITF treatments increased the proportion of acetate- and butyrate-producing bacteria, respectively, improved colonic inflammation and integrity, endotoxemia, and decreased helper T (Th)17 proportion in mesenteric lymph nodes (MLNs), blood, and aorta in SLE mice. However, disease activity (splenomegaly and anti-ds-DNA) was unaffected by both fibers. T cell priming and Th17 differentiation in MLNs and increased Th17 infiltration was linked to aortic endothelial dysfunction and hypertension after inoculation of fecal microbiota from SLE mice to GF mice, without changes in proteinuria and autoimmunity. All these effects were lower in GF mice after fecal inoculation from fiber-treated SLE mice. In conclusion, these findings support that fiber consumption prevented the development of hypertension by rebalancing of dysfunctional gut-immune system-vascular wall axis in SLE.

## Introduction

Systemic lupus erythematosus (SLE) is a highly deleterious autoimmune inflammatory disease linked to a higher risk of developing renal and cardiovascular complications.^1^ A combination of risk factors such as hypertension, dyslipidemia, and a prothrombotic state is implicated in the increased risk of cardiovascular disease in SLE. Increased atherosclerosis has already been shown in SLE. In addition to atherosclerosis, another underlying cause of cardiovascular disease in SLE is increased antiphospholipid antibodies, which can cause direct pro-inflammatory and prothrombotic effects on the endothelium, and interfere with the coagulation by inhibiting annexin A5 from its antithrombotic and protective effects.^1^ Specifically, SLE patients tend to develop hypertension with a high prevalence.^2^ Several factors (genetic, metabolic, hormonal, and environmental) play a role in the onset of SLE by boosting a chronic inflammatory response, leading to changes in blood pressure (BP).^3^ Nonetheless, the pathophysiological mechanisms behind SLE-linked hypertension are yet to be discovered. Interestingly, enhanced vascular reactive oxygen species (ROS) generation, which decreased nitric oxide (NO) bioavailability inducing endothelial dysfunction and vascular inflammation,^4^ has been involved on SLE hypertension.^5^

New studies on the subject have shown that gut bacteria can regulate SLE onset. Gut microbiota promotes the progression of SLE in both human and mouse models of SLE, mainly by promoting the development of a wide range of symptoms.^6–16^ Interestingly, using broad-spectrum antibiotics and fecal transplantation to recipient germ-depleted or germ-free mice, we demonstrated that gut microbiota contributed to the increase in blood pressure in both female NZBWF1 (F1 hybrid of New Zealand Black and New Zealand White strains) mice,^17^ and in BALB/cByJRj mice with SLE-induced by Toll-like receptor (TLR)7 activation.^18^ Relevant pioneer work demonstrated the role of gut microbiota on vascular function, showing that gut microbiota facilitated angiotensin II-induced vascular dysfunction and hypertension, at least in part, by vascular immune cell infiltration and IL-17-driven inflammation.^19^ Moreover, moderate high salt challenge in a pilot study in humans reduced intestinal survival of *Lactobacillus spp*. along with increased Th17 cells and BP, showing a gut-immune axis communication.^20^ In agreement with these data, priming of naive Th cells to Th17 in gut secondary lymph nodes and Th17 infiltration in the vasculature modulated by gut bacteria from lupus mice seem to be key events involved in vascular inflammation, endothelial dysfunction, and high BP in experimental models of SLE.^17,18^ These studies highlighted microbiota as a crucial target in the intervention of cardiovascular complications associated with SLE. In fact, the modulation of the gut microbiota composition by chronic consumption of the immune-modulatory bacteria *Lactobacillus fermentum* CECT5716 prevented vascular disorders^21^ and renal damage in the NZBWF1 mouse model of SLE.^22^

Diet is a major determinant of the gut microbiota composition. Western lifestyle is linked to autoimmune and metabolic diseases, driven by changes in diet and gut microbiota composition. Western diet is characterized by low dietary fiber intake. Some dietary fibers can be considered prebiotic if they are not digested in the upper gastrointestinal tract, passing intact to the large intestine, where they can be used by commensal bacteria in catabolic processes. Fermenting these prebiotics releases metabolites such as short-chain fatty acids (SCFAs, e.g. acetate, propionate, and butyrate). Two fermentable dietary-fibers of particular interest in this respect are resistant-starch (RS) and inulin. RS is an insoluble type of cereal fiber while inulin is a soluble fiber that can be found in many types of plant foods. The patients with SLE reported a lower fiber intake than healthy humans.^23,24^ Moreover, an inverse association between dietary fiber intake and the risk of active SLE has been described.^25^ In agreement with this association, Zegarra-Ruiz *et al*.^15^, using TLR7-dependent mouse models of SLE, found that a diet high in RS leads to greater SCFAs production to restrict growth of *Lactobacillus reuteri*, reducing the translocation from the gut to distal organs, and rescues lupus-prone mice from autoimmunity. Interestingly, gut bacteria thriving in the absence of prebiotic fiber is prohypertensinogenic,^26^ and supplementation of RS or SCFAs in diet prevented the rise of BP in rodents without genetic background of SLE.^26–32^ However, there is no information about the role of dietary fiber in cardiovascular complications in mice with genetic susceptibility to SLE. The aims of the present study were, therefore, to investigate the role of dietary fiber intake in the raise of BP in NZBWF1 mice and to explore the possible underlying mechanisms. Like in humans with SLE, the NZBWF1 mice produce anti-ds-DNA antibodies, develop immune complex glomerulonephritis, and, crucially, they develop hypertension.^33^ Considering the protective role of SCFAs in BP control, we used two types of fiber, inulin-type fructans (ITF) and RS, with high capacity of fermentation by the gut microbiota.

## Results

### Fiber treatments prevented the increase in blood pressure, targeting organ hypertrophy, renal injury but not disease activity in lupus-prone mice

**Figure 1.**
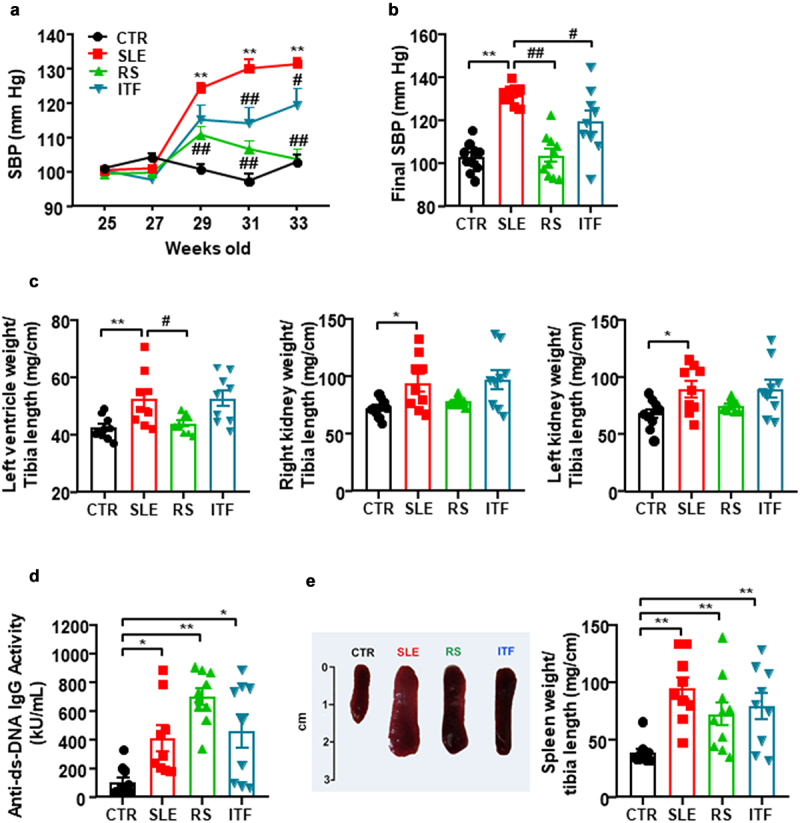
Fiber treatments inhibited the increase of blood pressure, target organ hypertrophy but not disease activity in systemic lupus erythematosus (SLE) mice.

Notes: (a) Time-course of systolic blood pressure (SBP) (n = 9-10, data are shown as means ± SEM, **P < 0.01 compared to the CTR group, ^#^*P* <0.05 and ^##^*P* <0.01 compared to the untreated SLE group, two-way ANOVA, Sidak’s multiple comparisons test) and (b) final SBP measured by tail-cuff plethysmography, (c) morphological parameters, (d) circulating double-stranded DNA autoantibodies levels, and (e) splenomegaly in control (CTR), SLE and SLE-groups treated with resistant starch (RS) or inulin-type fructans (ITF). Values are expressed as means ± SEM, n = 9-10, *P < 0.05 and **P < 0.01 compared to the CTR group, ^#^*P* <0.05 and ^##^*P* <0.01 compared to the untreated SLE group, one-way ANOVA.

The mouse mortality rate in each group was the following: CTR group, 0%; SLE group, 10%, 1 dead mouse out of 10; RS group, 0%; and ITF group, 10%, 1 dead mouse out of 10. At the end of the experiment, a significant increase in the body weight of SLE mice in comparison with body weight of CTR animals was found, and neither RS nor ITF significantly changed body weight in SLE mice (Figure S1A). In addition, no change in drink, food, and energy intake were observed among all experimental groups (Figure S1B). At 25 weeks of age, systolic blood pressure (SBP) values were similar for all experimental groups. At 33 weeks of age, we detected the characteristic rise in SBP in SLE by approximately 29 mmHg from CTR values, which was partially prevented by ITF (≈43%, *P* < 0.05) and totally by RS (*P* < 0.01) (Figure 1a, b). Sustained high blood pressure is one of the most powerful determinants of the development of cardiac and renal hypertrophy.^34^ Absolute heart weight and left ventricle weight were higher in SLE than in CTR group (166.7 ± 9.8 mg *vs*. 132.5 ± 3.6 mg, *P* < 0.001; 121.0 ± 7.6 mg *vs*. 95.5 ± 2.5 mg, *P* < 0.01, respectively), which were unaffected by both fiber treatments. Left ventricle weight/tibia length and right and left kidney weight/tibia length indices were increased in SLE compared to CTR values (≈21%, ≈ 30%, and ≈ 31%, respectively) (Figure 1c). RS treatment suppressed the observed cardiac hypertrophy but was unable to change the SLE high renal index. ITF did not change these morphological parameters (Figure 1c). SLE disease activity was determined at the experimental endpoint measuring plasma levels of anti-dsDNA autoantibodies, which revealed higher levels in SLE in relation to CTR (Figure 1d), as previously reported.^5,17^ Both fiber treatments did not significantly change disease activity, compared to SLE group. Likewise, splenomegaly has been used as a marker of disease progression, which can be associated with the development of a lymphoproliferative disorder.^35^ We have also detected this phenotypic characteristic in SLE (increase ≈2.4 times in spleen weight/tibia length compared to CTR group), and neither RS nor ITF treatments were able to change splenomegaly compared to SLE mice (Figure 1e).

**Figure 2.**
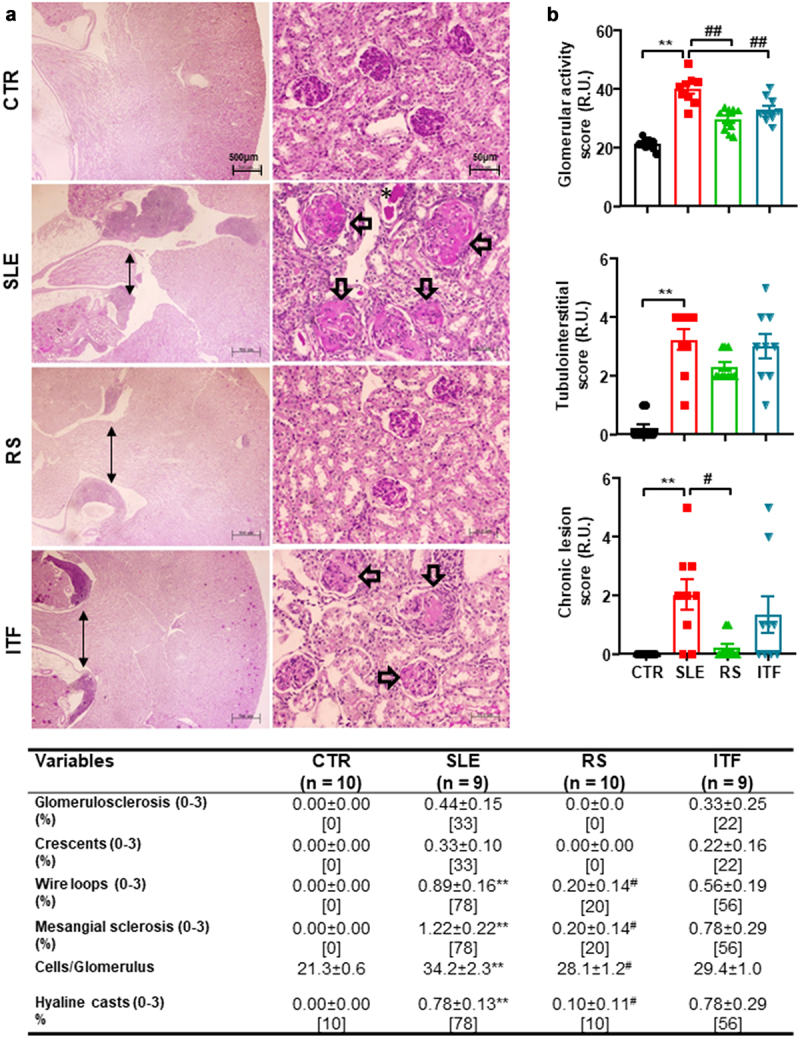
Fiber treatments improved morphological renal cortex features in systemic lupus erythematosus (SLE) mice.

Notes: (a) Kidney sections in control (CTR), SLE and SLE-groups treated with resistant starch (RS) or inulin-type fructans (ITF) were stained with hematoxylin-eosin and representative images are shown. Bar scale: 500 μm (left panels). Kidney sections were stained with periodic acid-Schiff and glomerular representative images are shown. Bar scale: 50 μm (right panels). Chronic inflammatory infiltrate in medullary area of kidney (black arrows), mesangial matrix expansion of glomeruli (white arrows), and hyaline tubular casts (asterisk). Table shows the quantification of renal lesions. Values are expressed as means ± SEM of percentage of affected glomeruli (n = 50/mouse). The percentage of mice with lesion is expressed in brackets [mice %]. (b) The mean scores for individual pathological features were summed to obtain the three main scores (the glomerular activity score, the tubulointerstitial activity score, and the chronic lesion score). Values are expressed as means ± SEM of relative units (R.U.). **P < 0.01 compared to the CTR group, ^#^*P* <0.05 and ^##^*P* <0.01 compared to the untreated SLE group,one-way ANOVA.

Renal injury is one of the characteristics most frequently associated with kidney inflammation in SLE.^5^ The main evidence for differences in renal injuries, their analyses, and representative images to illustrate them are shown in Figure 2. The kidneys of mice in the CTR group showed no remarkable glomerular, vascular, or tubulointerstitial lesions. Morphological examination of the glomeruli in SLE mice group revealed variable grades of intracapillary proliferation, mesangial sclerosis, wire loops, hyaline thrombi as well as very scan extracapillary proliferation (cellular crescent), fucsinophils deposits, and fibrous crescent formation. The quantification of glomerular cells showed an increased number of cells per glomerulus in SLE mice compared with CTR mice, which were reduced by RS supplementation but not ITF diet. Diffuse and global mesangial sclerosis, and intracapillary proliferation were significantly higher in SLE and ITF mice groups compared with RS mice and CTR groups. Glomerular activity scores were increased ≈ 5.8 fold in SLE as compared to CTR mice and were reduced by both types of diet (Figure 2b). In tubulointerstitial in renal papilla is evident, a moderate intensity of Tertiary Lymphoid Structures (TLS) (Figure 2a) and scan tubular casts. Kidney TLS has a similar cell composition, structure, and gene signature as lymph nodes and therefore may function as a kidney-specific type of lymph node.^36^ Lymphatic tissue swelling is one of the characteristics of lupus mice and is associated with SLE activity.^37^ TLS in renal papilla were unchanged by both RS and ITF (Figure 2a). Tubulointerstitial scores were ≈ 16-fold higher in SLE than in CTR group but were unchanged by both types of diet (Figure 2b). The group of SLE mice shows glomerular chronic lesions (Figure 2a), which increased the chronic lesion score (Figure 2b). The RS diet significantly reduces glomerular lesions, while the use of ITF diet does not modify the lesions.

### Fiber treatments-induced remodeling of gut microbiota composition

CTR and SLE did not present significant changes at the end of the experiment 1 for Chao richness (estimating the total operational taxonomic units in each given community), Pielou evenness (showing how individuals in the community are distributed over different operational taxonomic units), and Shannon diversity (that combines richness and evenness) (Figure S2A). We performed a two- and three-dimensional PLS-DA of the bacterial community, which measures microorganism diversity among samples, that is, β-diversity, at the level of the different taxa (phylum, class, order, family, genus, and species), in an unsupervised manner. This analysis showed a no significative clustering of the animals into the CTR and SLE groups. However, well-separated populations were seen for PLS-DA between the clusters for SLE *vs* SLE-RS or SLE *vs* SLE-ITF (Figure S3A). According to the VIP score, prominent changes in bacterial genus occurred among groups (Figure S3B). We did not detect changes in the proportion of bacteria from several phyla (Figure S2B). Firmicutes/Bacteroidetes (F/B) was also unchanged (Figure S2C). ITF supplementation to SLE mice increased evenness. However, RS diet induced more profound changes in phyla proportion, increasing Verrucomicrobia reads. Interestingly, both types of fibers also increased strict anaerobes bacteria content in feces (Figure S2D). At family level, reduced *Clostridiaceae* and increased *Lactobacillaceae* proportions were found in SLE as compared to CTR (Figure S4). RS treatment increased *Bacteroidaceae*, *Rikenellaceae*, and *Verrucomicrobiaceae* proportions (Figure S4), mainly due to a significative expansion of genera *Bacteroides* (*Bacteroidaceae*), *Parabacteroides* (*Porphyromanadaceae*), *Alistipes* (*Rikenellaceae)*, and *Akkermansia (Verrucomicrobiaceae)* (Figure S5). In addition, RS diet reduced *Lactobacillus* (*Lactobacillaceae*), *Barnesiella* (*Porphyromanadaceae*), and *Roseburia (Lachnospiraceae)*. By contrast, the changes induced by soluble fiber focus mainly on inducing an expansion of *Clostridiaceae*, especially *Clostridium* genus, and a contraction in *Bacteroidaceae* (*Bacteroides* genus), *Lactobacillaceae* (*Lactobacillus* genus), *Bacillaceae*, and *Roseburia*. Species, such as *Akkermansia muciniphila* and *Bacteroides acidifaciens*, were increased by RS treatment. Both fiber treatments were unable to change *Lactobacillus reuteri* content (Figure S6).

**Figure 3.**
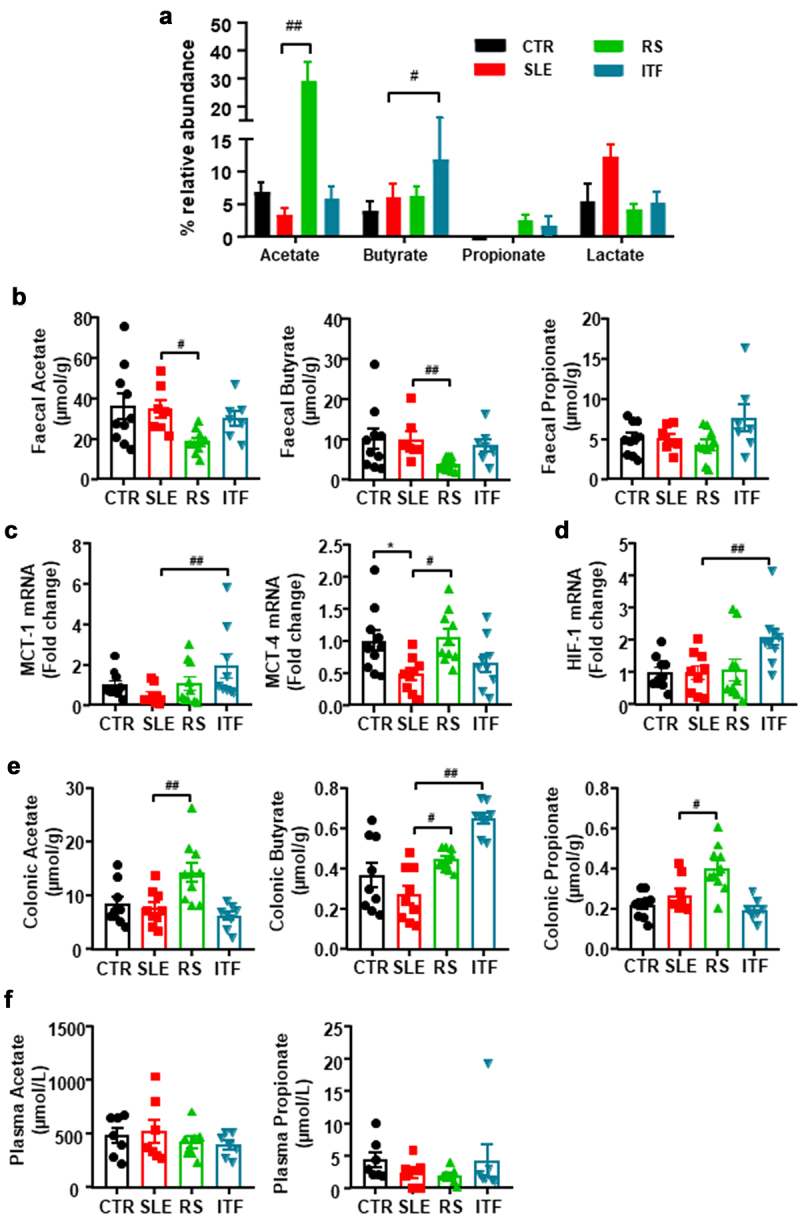
Fiber treatments changed short chain fatty acids (SCFAs) bioavailability in systemic lupus erythematosus (SLE) mice.

Notes: (a) Proportion of SCFAs producing- bacteria in feces from control (CTR), SLE and SLE-groups treated with resistant starch (RS) or inulin-type fructans (ITF) measured by 16S rRNA analysis (n = 8-10). (b) Concentrations of SCFAs in feces from all experimental groups measured by HPLC-ESI-MS and expressed as µmol/g of lyophilized feces. (c) Colonic mRNA levels of monocarboxylate-transporter (MCT)1 and MCT4, and (d) hypoxia inducible factor (HIF)-1. (e) Concentrations of SCFAs in colonic tissue expressed as µmol/g of lyophilized colon, and (f) in plasma from all experimental groups measured by HPLC-ESI-MS and expressed as µmol/L. Values are expressed as means ± SEM, n = 9-10, *P < 0.05 compared to the CTR group, ^#^*P* <0.05 and ^##^*P* <0.01 compared to the untreated SLE group, one-way ANOVA.

Considering that fermentation of prebiotic fiber by the gut bacteria leads to the production of SCFAs, we analyzed the relative abundance of SCFAs-producing bacteria. As described previously,^17^ no significant changes in SCFAs-producing bacteria were found between CTR and SLE. However, acetate-producing bacteria were increased by RS diet, whereas ITF increased the proportion of butyrate-producing bacteria (Figure 3a). When we measured SCFAs content in feces, we found reduced acetate and butyrate content in SLE-RS group as compared to SLE mice, being unchanged in ITF-treated mice (Figure 3b) suggesting increased absorption of SCFAs in fiber-treated groups. In general, bacteria-produced SCFAs may follow a colonic-hepatic-periphery distribution. Colonic levels were followed by a significant drop, around 10-fold, in the liver, reaching the periphery in the µM range.^38^ SCFA might be able to enter the cytosol by passive diffusion, but additionally they can be absorbed by solute transporters, such as the proton-coupled monocarboxylate-transporter (MCT)1 and MCT4,^39^ which are upregulated by chronic acetate or butyrate consumption.^32^ MCT1 is the principal transporter for butyrate in intestinal epithelial cells and it is upregulated by butyrate and fermentable carbohydrates.^40^ This justifies our findings by which ITF treatment increased butyrate-producing bacteria in SLE mice, also increased the colonic mRNA levels of MCT1, whereas RS, which increased acetate-producing bacteria, increased the mRNA levels of MCT4 (Figure 3c). Treatment with SCFAs induces a higher proliferative activity and turnover in GF or antibiotic-treated SPF mice.^41^ In agreement with this, we found that colonic weight/length ratio, an index of epithelial cells proliferation was similar between CTR and SLE group (14.4 ± 0.4 *vs*.15.5 ± 1.4 mg/cm, respectively, *P* > 0.05), but was ≈ 60% higher in RS-treated mice as compared to SLE group, showing promotion of intestinal epithelial cells turnover. Additionally, butyrate in intestinal epithelial cells consumes (local) O_2_, stabilizing the hypoxia inducible factor (HIF, a transcription factor coordinating barrier protection).^42^ We also found increased colonic HIF-1 mRNA levels in ITF-treated SLE mice as compared to SLE group (Figure 3d). In addition, increased contents of acetate, butyrate, and propionate in the RS group and butyrate in the ITF group as compared to SLE mice were detected in colon tissue (Figure 3e), possibly due to increased uptake. These SCFAs are absorbed from the gut into the hepatic portal circulation and/or lacteal lymphatic system to the liver. In the liver, lower butyrate and higher propionate levels were found in the RS group than in SLE group (Figure S7). Butyrate and propionate, mostly metabolized by hepatocytes, appear at low concentration in the systemic circulation.^43^ In agreement with this information and despite higher production and absorption of SCFAs in fiber-treated mice, we found similar plasma levels of acetate and propionate in all experimental groups (Figure 3f). Plasma level of butyrate was below the detection limit (0.2 μM) using a LC-QqQ-MS determination. Overall, our data demonstrated that fiber treatments induced modifications in gut microbiota composition characterized by increased acetate- or butyrate-producing bacteria. These SCFAs upregulate colonic MCTs transporters increasing their absorption into intestinal epithelial cells, leading to improved colonic homeostasis and reaching the liver, where they were metabolized resulting in similar plasma levels between groups.

### Fiber treatments improved intestinal integrity and inflammation

Given the importance of gut commensal translocation in autoimmunity,^14^ we next assessed the integrity of the intestinal epithelium. We studied the gut barrier integrity through colonic mRNA levels and protein expression of barrier-forming junction transcripts (Figure S8A), such as occludin and zonula occludens-1 (ZO-1) and the mucins (Figure S8B), mucin-2 (MUC-2), and MUC-3. Reduced mRNA levels of ZO-1, MUC-2 and MUC-3 were observed in SLE group as compared to CTR and treated groups. However, protein expression of occludin was lower in SLE than in CTR, being without change ZO-1. Consistent with this reduced intestinal integrity in SLE mice we found increased (≈5.4-fold) plasma lipopolysaccharide (LPS) levels (Figure S8C). In agreement with previous data,^15^ RS diet improved these markers of gut integrity and reduced plasma endotoxin levels. By contrast, ITF only significantly increase colonic occludin expression, but was unable to change endotoxemia. These results suggest that intestinal permeability is high for SLE mice allowing bacterial components (e.g., LPS) into the blood stream. Moreover, both fibers reduced mRNA levels of proinflammatory cytokines interleukin (IL)1ß and Tumour Necrosis Factor (TNF)α (Figure S8D). In epithelial and immune cells SCFAs behave like ligands for G-protein coupled receptors (GPCRs), such as GPR43, and GPR41, which are upregulated by SCFAs. Moreover, SCFAs (mainly butyrate) have direct inhibitory effects over histone deacetylases (HDACs) activity triggering histone acetylation, modulating gene regulation of cell proliferation, differentiation, and the inflammatory response, contributing to intestinal homeostasis.^40^ In agreement with this, we found higher GPR43 and GPR41 mRNA level in colonic samples from RS and ITF groups, respectively, as compared to SLE mice (Figure S8E). Moreover, colonic HDAC3 transcript was downregulated in the ITF group as compared to SLE mice (Figure S8E). Overall, our results are consistent with increased colonic integrity and reduced inflammation induced by fiber treatments, linked to GPR43 activation in RS-treated mice, and GPR41 activation and HDAC inhibition in ITF-treated mice.

### Fiber treatments attenuated T cells imbalance

**Figure 4.**
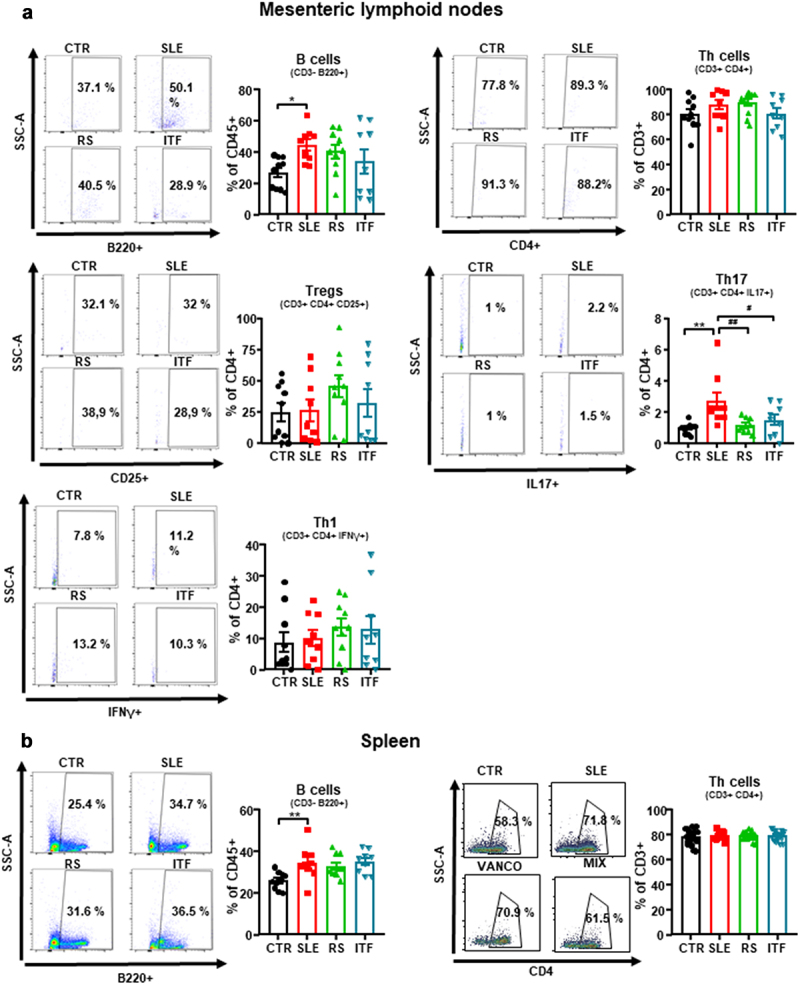
Effects of fiber treatments on lymphocytes populations in systemic lupus erythematosus (SLE) mice.

Notes: (a) Total B lymphocytes, T helper (Th) cells, Regulatory T cells (Treg), Th17, and Th1 cells measured by flow cytometry in mesenteric lymphoid nodes, and (b) B and Th cells in spleen from control (CTR), SLE and SLE-groups treated with resistant starch (RS) or inulin-type fructans (ITF). All data are expressed as % of parent, except for B cells, that are represented as % of grandparent (% of CD45+). Values are expressed as means ± SEM, n = 9-10, *P < 0.05 and **P < 0.01 compared to the CTR group, ^#^*P* <0.05 and ^##^*P* <0.01 compared to the untreated SLE group, one-way ANOVA.

Increased autoantibody production and lupus-like autoimmune disease progression are associated with a T cell imbalance and high B cells levels.^44,45^ We assessed B and T cell populations from mesenteric lymph nodes (MLNs), spleen, and blood. Levels for B cells were increased in the secondary lymphoid organs from SLE compared to CTR. Meanwhile, T cells were unchanged (Figure 4a, b). Fiber treatments produced no changes on the levels of B and Th cells in either lymphatic organ. In conditions of disrupted gut mucosal integrity, such as those found in SLE, bacteria translocate through the intestinal barrier triggering the activation and migration of CX3CR1+ cells, such as dendritic cells or macrophages, toward drain lower intestinal tract lymph nodes.^46^ These cells additionally present antigens to naïve CD4+ T lymphocytes, leading to T cell priming. In correlation with the results of intestinal integrity described above, we found high levels of CX3CR1 mRNA in MLNs from SLE group as compared CTR mice, which were reduced by RS treatment, but not by ITF (Figure S9A). Dendritic Cells (DC) from hypertensive mice CD80^high^ and CD86^high^ (common B7 ligands), which points to DC maturation and activation.^47^ In our experiment, MLNs from SLE mice showed higher CD80 and CD86 mRNA levels as compared to CTR mice, and only RS treatment restored their levels like CTR group (Figure S9B). MLNs T lymphocytes upregulate integrinα4β7.^48^ We observed that Itga4 but not Itgb7 expression was higher in MLNs from SLE mice than CTR group (Figure S9C), pointing to an increased activation of T cells. RS consumption reduced both integrin subunits, showing reduced T cells activation. Beyond their role as antigen presenting cells, DCs release mediators promoting T cell polarization. IL‐6 induces Th17 cell proliferation and inhibits Treg cell differentiation.^49^ We analyzed its transcript levels in MLNs and observed that were significantly augmented in SLE group when compared to those found in CTR group and were only normalized by RS treatment (Figure S9D). In consequence, the percentage of Th17 cells (CD4+/IL-17a+) increased ≈ 2.8-fold in SLE mice in MLNs (Figure 4a), being Treg (Treg, CD4+/CD25+) and Th1 (CD4+/IFN-γ+) unchanged. Interestingly, both RS and ITF treatments normalized Th17 content, suggesting that ITF exert immunoregulatory effect independently of IL-6 levels. Consistent with higher acetate production and absorption in RS group, GPR43 level was increased by RS treatment in MLNs (Figure S9E). It has been described that sodium butyrate, acting as HDAC inhibitor, regulates Th17/Treg cell balance to ameliorate via the nuclear factor erythroid 2-related factor 2 (Nrf2)/heme oxygenase 1 (HO-1)/IL-6 receptor pathway.^50^ We found that mRNA levels of HDAC3 in MLN were reduced by ITF treatment, whereas RS mice were without effect (Figure S9F). Consistent with Nrf2 activation, the mRNA levels of the down-stream antioxidant enzymes HO1 and NAD(P)H:quinone oxidoreductase 1 (NQO1) mRNA levels were increased by ITF (Figure S9G), leading to reduced expression of IL-6 receptor (Figure S8H). When we analyzed Th17 and Treg cell content in colonic lamina propria by immunofluorescence, we found higher Th17 cells in SLE as compared with CTR mice, which was reduced by both RS and ITF treatments, whereas no significant changes in Treg content were observed among all experimental groups (Figure S10). In spleen, irrigated by systemic circulation, no significant changes in Treg, Th17, and Th1 were induced by RS or ITF treatment (Figure S11A). Circulating B, Treg, Th1, and Th17 cells were increased in SLE compared to CTR (Figure S11B). Following the trends observed in MLNs, RS and ITF diets decreased the proportion of circulating Th17 cells.

### Fiber treatments prevented endothelial dysfunction, vascular oxidative stress, and Th17 infiltration in aorta

**Figure 5.**
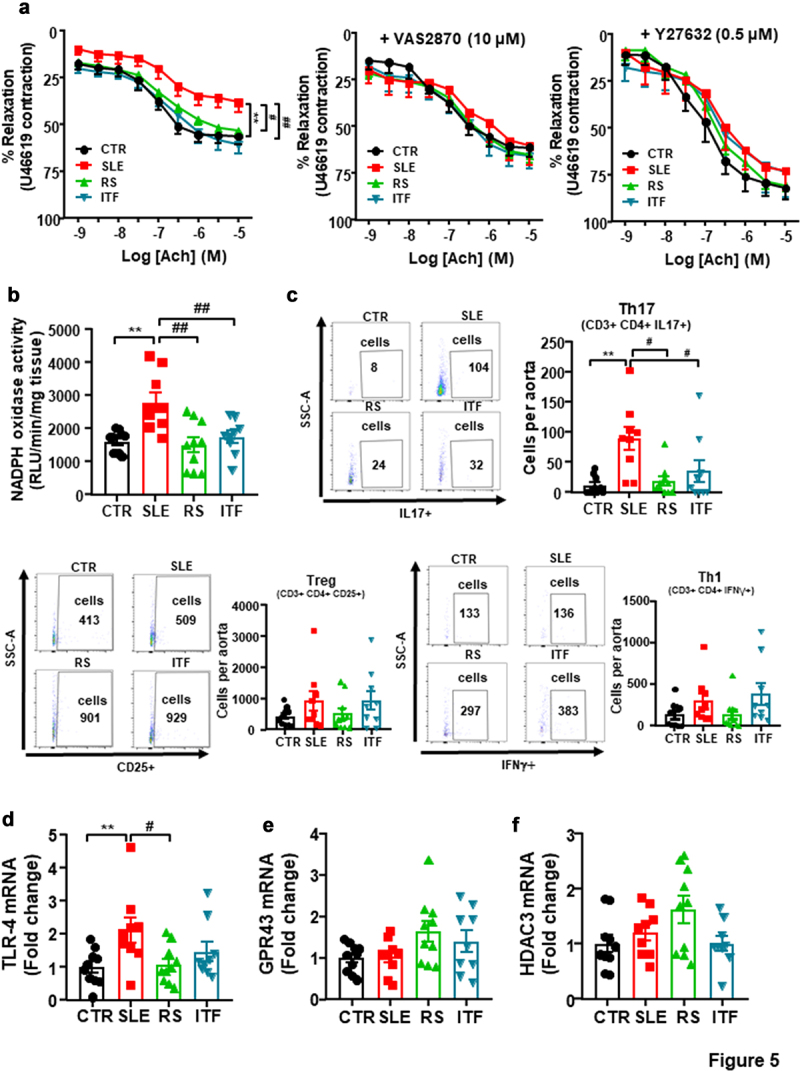
Fiber treatments improved endothelial function, NADPH oxidase activity and aortic infiltration of immune cells in systemic lupus erythematosus (SLE) mice.

Notes: (a) Vascular relaxation responses induced by acetylcholine (Ach) in endothelium-intact aortas pre-contracted by U46619 (3 nM), in the absence or in the presence of the NADPH oxidase inhibitor VAS2870 (10 µM) or the Rho kinase inhibitor Y27632 (0.5 µM) in all experimental groups (n = 9-10, data are shown as means ± SEM, *P < 0.05 compared to the CTR group, ^#^*P* <0.05 compared to the untreated SLE group, two-way ANOVA, Dunnett’s multiple comparisons test). (b) Aortic NADPH oxidase activity measured by lucigenin-enhanced chemiluminescence. (c) Aortic infiltration of immune cells measured by flow cytometry. (d) Aortic mRNA levels of toll-like receptor (TLR)4, G protein-coupled receptor (GPR)43, and histone deacetylase (HDAC)3. Groups: control (CTR), SLE and SLE-groups treated with resistant starch (RS) or inulin-type fructans (ITF). Values are expressed as means ± SEM, n = 9-10, **P < 0.01 compared to the CTR group, ^#^*P* <0.05 and ^##^*P* <0.01 compared to the untreated SLE group, one-way ANOVA.

SLE aortas showed diminished endothelium-dependent vasorelaxant responses to acetylcholine compared to CTR (Emax = 38.3 ± 5.4% and 56.4 ± 4.4%, respectively, *P* < 0.01) (Figure 5a). RS and ITF fibers improved the impairment of acetylcholine-induced relaxation. This acetylcholine-induced response was also improved in SLE after incubation with the pan-NOX inhibitor VAS2870 or the Rho kinase inhibitor Y27632 (Figure 5a), suggesting that the impairment in acetylcholine-induced relaxation is mediated, at least in part, by NADPH oxidase and Rho kinase activation. ROS-dependent activation of RhoA/Rho kinase has been previously described.^51^ NADPH oxidase is the main source of ROS in the vascular wall, we quantitated NADPH oxidase activity. NADPH oxidase activity (Figure 5b) was ≈ 1.7-fold higher in aortic rings from SLE than CTR group, and both type of fiber inhibited this activity. Considering that inflammatory cells boosted vascular ROS synthesis, we studied T lymphocyte extravasation in aorta. Th17 cells were higher in aorta from SLE as compared to CTR, we did not observe significative changes in Treg and Th1 cells (Figure 5c). Both RS and ITF treatments reduced the infiltration of Th17 in aorta. In addition, LPS stimulates and increases the expression of toll-like receptor (TLR)4 in the vasculature, which resulted in increased NADPH oxidase activity.^52^ In correlation with plasma LPS levels, mRNA levels of TLR4 were higher in SLE compared to CTR group, which were significantly reduced by RS treatment (Figure 5d). SCFAs reduced NADPH oxidase activity through activation of GPCRs or HDACs inhibition.^53^ However, no significant changes in aortic GPR43 (Figure 5e) or HDAC3 (Figure 5f) were found among all experimental groups, ruling out that a direct action of SCFAs in vascular wall was involved on the vasculo-protective effect induced by both fiber treatments.

To address the question whether changes in gut microbiota induced by fiber intervention in lupus mice play a role in their protective effects on gut, immune system, BP and endothelial function, we inoculated microbiota from all experimental groups to recipient normotensive female C57Bl/6J GF mice, which were maintained for 3 weeks.

### Fiber treatments abolished hypertensive phenotype induced by gut microbiota from female NZBWF1 mice in germ free mice

**Figure 6.**
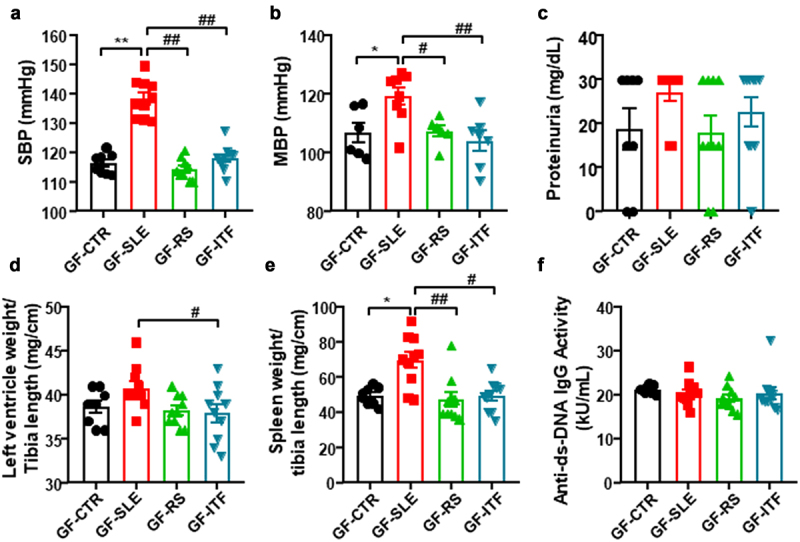
Fiber treatments prevented the transfer of hypertensive phenotype to germ-free mice induced by inoculation of feces from systemic lupus erythematosus (SLE) mice.

Notes: (a) Systolic blood pressure (SBP) measured by tail-cuff plethysmography. (b) Mean arterial blood pressure (SBP) measured by direct register in carotid artery. (c) Urine protein concentration measured by Combur test strips. (d) Left ventricular weight/tibia length ratio was measured as morphological parameter in the heart. (e) Spleen weight/tibia length ratio, and (f) autoantibody levels were measured as markers of the pathology. Groups: germ-free (GF) inoculated with control feces (GF-CTR), GF inoculated with SLE feces (GF-SLE) and GF inoculated with feces from SLE-groups treated with resistant starch (GF-RS) or with inulin-type fructans (GF-ITF). Values are expressed as means ± SEM, n = 8-10, *P < 0.05 and **P < 0.01 compared to the GF-CTR group, ^#^*P* <0.05 and ^##^*P* <0.01 compared to the GF-SLE group, one-way ANOVA.

As expected, donor SLE microbiota increased SBP in recipient GF mice to a maximum of ≈22 mmHg, as compared to fecal inoculation of donor CTR mice (Figure 6a). Interestingly, a significant reduction in SBP was observed in mice inoculated to SLE feces from mice treated with fibers, showing that changes in gut microbiota induced by fiber treatments disrupt the hypertensive phenotype of microbiota from SLE mice. Intra-arterial mean blood pressure (MBP) was recorded to confirm the effects of fecal transfer on BP (Figure 6b). However, SLE microbiota transplantation for 3 weeks was unable to evoke higher protein excretion (Figure 6c) or to induce significant morphological changes in left ventricle (Figure 6d). Interestingly, a low degree of splenomegaly was induced by gut microbiota from SLE mice (increase ≈41% in spleen weight/tibia length compared to GF-CTR group), which disappeared in GF-RS and GF-ITF mice (Figure 6e). Despite this, no significant changes in plasma levels of anti-dsDNA were observed among all experimental groups (Figure 6F), showing no change in lupus activity induced by microbiota from SLE mice.

### Fiber treatments inhibited the impaired gut integrity and immune imbalance induced by gut microbiota from female NZBWF1 mice in germ-free mice

Inoculation of SLE microbiota to GF mice reduced colonic mRNA levels of ZO1, without significant changes in occludin (Figure S12A), MUC-2 and MUC-3 (Figure S12B). Gut microbiota transplantation from SLE-RS group to GF mice increased occludin, ZO-1 and mucins mRNA levels, whereas feces from SLE mice treated with ITF fiber showed similar profile than untreated SLE mice. Plasma LPS levels were increased slightly (≈34%) in GF-SLE group as compared to GF-CTR group (Figure S12C). Moreover, microbiota inoculation from RS and ITF to GF mice reduced colonic mRNA levels of TNFα and IL1ß expression (Figure S12D). Interestingly, the SCFAs receptor GPR43 transcript was increased GF-RS (Figure S12E), whereas mRNA levels of HDAC3 were reduced in GF-ITF as compared to GF-SLE (Figure S12F), showing a role of SCFAs in the colonic effects of the microbiota from fiber-treated SLE mice inoculated into GF, despite GF mice-fed standard diet.

In MLNs, the higher transcript levels of CD80, Itga7, and IL-6 found in GF inoculated with SLE microbiota, as compared to mice inoculated with feces from CTR group, were normalized in GF-RS group (Figure S13A). By contract, no change was observed in GF-ITF mice (Figure S13A). These data showed that protective effects of RS fiber on T cell activation in gut secondary lymph nodes were transferred by the microbiota. Moreover, GPR43 and HDAC3 expression were increased in GF-RS and reduced in GF-ITF, respectively (Figure S13B), involving SCFAs. Nrf2 activation was detected in GF-ITF group, since HO-1 and NQO1 mRNA levels were higher than GF-SLE group, and IL-6 R expression where lower (Figure S13C). According to IL-6 or IL-6 R levels, the transplantation of microbiota from SLE to recipient GF mice increased the Th17 proportion in MLNs as compared to CTR microbiota transfer (Figure S14A). Interestingly, reduced proportion of Th17 population was found in MLNs and blood from GF-RS and GF-ITF groups, as compared to GF-SLE mice, being unaltered in spleen (Figure S14A-C). Other changes in B, and Tregs cells induced by SLE microbiota inoculation were unaffected by both fiber interventions (Figure S14A-C). Overall, our data demonstrated that fiber treatments restored Th17/Treg balance in SLE mice, at least in part, by changing gut microbiota composition.

### Fiber treatments abolished gut microbiota-induced endothelial dysfunction in female NZBWF1 mice in germ-free mice

**Figure 7.**
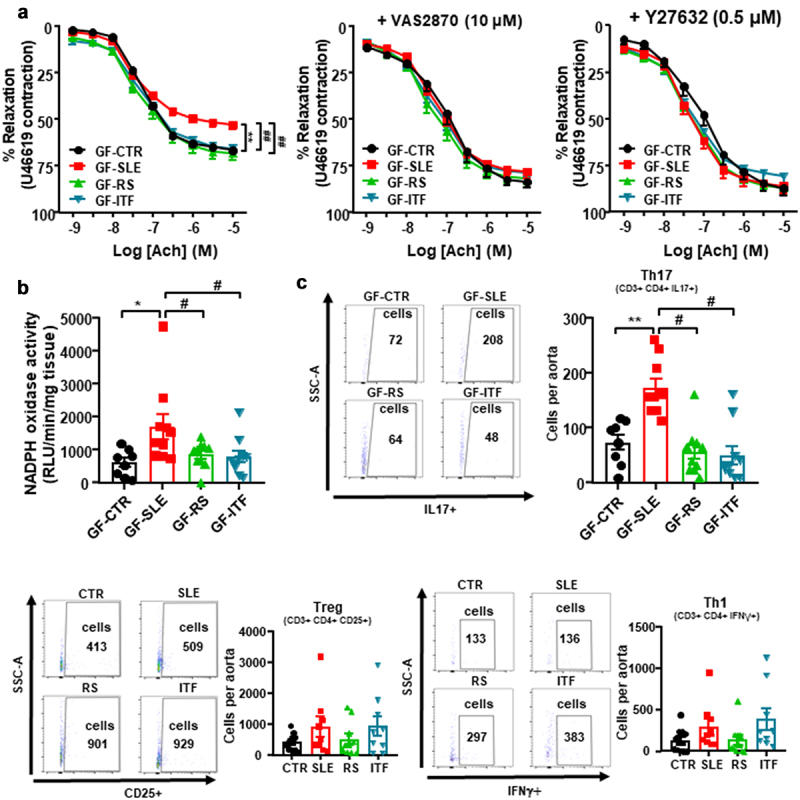
Fiber treatments prevented the transfer of endothelial dysfunction phenotype to germ-free mice induced by inoculation of feces from systemic lupus erythematosus (SLE) mice.

Notes: (a) Vascular relaxation responses induced by acetylcholine (Ach) in endothelium-intact aortas pre-contracted by U46619 (3 nM), in the absence or in the presence of the NADPH oxidase inhibitor VAS2870 (10 µM) or the Rho kinase inhibitor Y27632 (0.5 µM) in all experimental groups (n = 8-10, data are shown as means ± SEM, **P < 0.01 compared to the CTR group, ^##^*P* <0.01 compared to the untreated SLE group, two-way ANOVA, Dunnett’s multiple comparisons test). (b) Aortic NADPH oxidase activity measured by lucigenin-enhanced chemiluminescence. (c) Aortic infiltration of immune cells measured by flow cytometry. Groups: germ-free (GF) inoculated with control feces (GF-CTR), GF inoculated with SLE feces (GF-SLE) and GF inoculated with feces from SLE-groups treated with resistant starch (GF-RS) or with inulin-type fructans (GF-ITF). Values are expressed as means ± SEM, n = 8-10, *P < 0.05 and **P < 0.01 compared to the GF-CTR group, ^#^*P* <0.05 compared to the GF-SLE group, one-way ANOVA.

Endothelium-dependent relaxant curves to acetylcholine in U46619-precontracted GF-SLE aortas were highly impaired when compared to GF-CTR group (Emax: 52.8 ± 2.9% *vs*. 66.6 ± 1.1%, *P* < 0.01, respectively; Figure 7a), showing that endothelial dysfunction found in SLE mice was, at least in part, mediated by gut microbiota, and that this vascular phenotype was transferred to mice without SLE background by microbiota inoculation. However, this impaired of acetylcholine relaxation was absent in aorta from GF mice inoculated with feces from SLE mice treated with RS or ITF. Incubation for 30 min with the pan-NOX inhibitor VAS2870 or the Rho kinase inhibitor Y27632 abolished differences between groups in relaxation to acetylcholine, showing the involvement of NADPH oxidase and Rho kinase in this impaired relaxant response induced by SLE microbiota (Figure 7a). In fact, the fecal transplant from SLE caused an increase in aortic NADPH oxidase activity (Figure 7b), as compared to CTR microbiota inoculation. Remarkably, Th17 infiltration in aorta was higher in GF-SLE than GF-CTR group, being without changes in Tregs and Th1 (Figure 7c). Again, inoculation with feces from SLE mice treated with RS or ITF reduced both aortic NADPH oxidase activity and Th17 infiltration. Overall, our data showed that fiber treatments improved vascular oxidative stress and endothelial dysfunction in SLE mice by inducing changes in gut microbiota which led to reduced vascular Th17 infiltration.

## Discussion

The most important information from this study is that preventive cardiovascular effects of fiber interventions in SLE mice were associated with the rebalancing of dysfunctional gut-immune system-vascular wall axis. This was supported by several pieces of evidence: (i) increasing SCFAs producing bacteria; (ii) normalization of gut integrity and leakiness; (iii) reduction of Th17 polarization in MLNs and lamina propria and lower vascular Th17 infiltration; (iv) dampened endothelial dysfunction and high BP, and (v) feces inoculation from SLE mice treated with RS or ITF to GF transferred the improved gut-immune system-vascular wall axis.

Gut microbiota-host genetics interaction plays an important role in the progression of autoimmune diseases, like SLE. It has been previously demonstrated that gut microbiota and gut-immune system communication are crucial in the development of endothelial dysfunction and hypertension in female NZBWF1 mice.^17^ We must highlight that the PLS-DA of gut microbiota in NZBWF1 mice and age-matched control mice did not demonstrate highly differentiated microbial communities. In agreement with previous evidence,^17,21^ the main characteristics of gut microbiota remodeling in SLE were: (i) No significant changes in α-diversity parameters (richness, diversity, and evenness), in F/B ratio, in SCFAs-producing bacteria and in strict anaerobic bacteria proportion; (ii) The main changes happen within the sublevel categories as family and genera, with reduced content in *Clostridiaceae* and increased *Lactobacillus* (*Lactobacillaceae*). NZB/WF1 mice displayed a higher abundance of *Lactobacilli* in the gut microbiota, which may be associated with more severe clinical signs, especially the impairment of systemic autoimmunity and renal function.^9^ Our results agree with the key role of *Lactobacilli* in the development renal dysfunction in SLE since RS and ITF interventions, which reduce *Lactobacillus* proportion, improved renal injury. Interestingly, the fecal content of *Lactobacillus reuteri*, a SCFAs-sensitive bacteria, and its translocation to secondary lymph nodes and liver seems to be involved in SLE autoimmunity.^15^ However, in our experimental conditions, the proportion of *L. reuteri* in feces from CTR and SLE mice was similar and was unaffected by either RS or ITF, suggesting that translocation of this bacteria is not involved in autoimmunity in NZB/WF1 mice.

Prebiotic fiber fermentative metabolization by the gut microbiota produces metabolites known as SCFAs (e.g. acetate, propionate, and butyrate), which have been demonstrated to be relevant regulators of pro-hypertensive components in SLE, such as inflammatory and immune processes.^54,55^ As expected, fiber supplementation promoted the growth of acetate-producing bacteria (RS fiber) and butyrate-producing bacteria (ITF fiber). *Bacteroides acidifacients* (*Bacteroidaceae*, phylum Bacteroidetes), an acetate-producing bacteria, which was associated with reduced BP in hypertensive animals, was increased by RS fiber consumption.^27^

SCFAs are relevant metabolites for the maintenance of intestinal homeostasis. SCFAs can act as fuel for intestinal epithelial cells and intervene in the strengthening of the gut barrier function.^40^ In our experimental conditions, reduced colonic integrity was found in SLE mice, associated with increased translocation of LPS into the circulation. RS increased the expression of tight-junction proteins and reduced endotoxemia. Interestingly, colonic upregulation of SCFAs receptor GPR43 was induced by RS fiber, suggesting that binding of acetate to GPR43 is a possible mechanism to improve gut integrity in SLE, whereas butyrate-induced HDAC inhibition seems to be involved in the protective effect of ITF fiber. In addition, RS fiber significantly increased colonic mRNA levels of mucins, associated with a significant increase of *Akkermansia muciniphila*. This is a gram-negative, strictly anaerobic bacterium belonging to the Verrucomicrobia phylum. It is capable of degrading mucin. These mucolytic properties seem to stimulate mucus renewal by a positive feedback loop.^56^ In addition, *A. muciniphila* was associated with improved endothelial dysfunction, an early marker of cardiovascular disease, in apolipoprotein E knockout (Apoe^−/−^) mice,^57^ and reduced endotoxemia.^58^

Recent publications that study female NZBWF1 mice have demonstrated that there is a broad arrange of factors playing a role in the onset of hypertension besides B-cell hyperactivity and autoantibody synthesis, such as pro-inflammatory cytokines or oxidative stress.^54^ These are elements that mainly mediate local inflammation and can be linked to renal and vascular dysfunction. They can be found with a high probability downstream of the initial genetically determined immune system dysregulation.^55^ Previous studies demonstrated that dysfunctional polarization of naïve T cells to Th17 in MLNs and Th17 infiltration in the vascular wall are crucial events involved in the gut microbiota-mediated higher BP in SLE mice.^17,21^ The present study agrees with this hypothesis and demonstrated that fiber treatments reshaped gut-immune system axis, reducing Th17 polarization in MLNs. In fact, stool inoculation from SLE mice treated with RS or ITF was unable to increase Th17 content in GF MLNs as compared to fecal inoculation from untreated SLE mice. SCFAs, such as acetate or butyrate, might mediate this gut-immune system communication in this part of the intestine, as previously described.^32^ As expected, B cell populations were higher in secondary lymph organs from SLE mice as compared to CTR in our results, and neither RS nor ITF treatments reduced B cell generation and circulating B cells, discarding the involvement of B cells in the BP regulation induced by microbiota. Likewise, fecal microbiota transplant from SLE mice to GF mice did not increase the proportion of B cells in MLNs. As per these results, we did not gather sufficient evidence to prove a hypothetical pathogenic role of anti-ds-DNA, as mediator of BP increase induced by microbiota. In fact, both fiber treatments decreased BP but were not able to reduce plasma anti-ds-DNA. Also, fecal inoculation from hypertensive SLE mice to GF mice induced an increase in BP but could not alter circulating anti-ds-DNA.

Endothelial dysfunction plays a seminal part in the pathogenesis of hypertension. Decreased NO bioavailability is the central factor that links oxidative stress to endothelial dysfunction and hypertension. Both innate and adaptive immune responses participate in the generation of ROS and inflammatory changes in the kidneys, blood vessels and brain in hypertension.^59^ A dysfunctional communication between immune system and vascular wall is involved in endothelial dysfunction in SLE mice.^21,60^ High NADPH oxidase-driven ROS synthesis is linked to both endothelial dysfunction and high BP in female NZB/WF1 mice.^5,17,21^ Accordingly, we too have detected a reduction in acetylcholine-induced relaxation and an increase in NADPH oxidase activity in aorta from SLE as compared to CTR. It is interesting that chronic fiber treatments were able to prevent the impoverished responses to acetylcholine and the increase in NADPH oxidase activity. We were able to corroborate these effects through fecal microbiota transplantation to GF mice, involving gut microbiota in oxidative stress and endothelial dysfunction. ROS production by the vascular NADPH oxidase has been seen as a crucial part of microbiota-induced endothelial dysfunction since incubation with the selective NADPH oxidase inhibitor VAS2870 suppresses the impairment of aortic endothelium-dependent relaxation to acetylcholine. Local and circulating cytokines can modulate NADPH oxidase activity.^21,61,62^ Both RS and ITF decreased Th17 maturation in MLNs, circulation and vascular infiltration (as seen in aorta). It has already been established that the pro-inflammatory cytokine IL-17 induces Rho-kinase-mediated endothelial dysfunction in the vasculature,^63^ presumably partially because of an increase in ROS generation by NADPH oxidase activation.^64^ Thus, the Rho-kinase inhibitor Y27632 restored acetylcholine relaxation similarly to what we observed with the fiber treatments, which suggest that the IL-17-Rho-kinase-pathway is highly regulated by gut microbiota in our genetic SLE model. Additionally, stool inoculation from SLE mice treated with RS or ITF to GF was unable to induce Th17 populations in MLNs, Th17 infiltration in aorta and impaired acetylcholine relaxation, as compared to fecal inoculation from untreated SLE mice. Overall, fibers intervention improved immune system-vascular axis, reducing Th17 polarization in secondary lymph nodes and restoring endothelial function in SLE mice.

Activating TLR-4 in vessels with bacterial products like LPS increases NADPH oxidase-dependent O_2_^−^ production and inflammation.^52^ In SLE mice, plasma endotoxin levels were increased, and intervention addressed to reduce endotoxemia normalized vascular TLR-4 expression and improved both vascular oxidative stress and inflammation.^17,21^ In addition, we were able to find increased LPS plasma levels in SLE mice associated with lower colonic integrity. Fiber interventions, especially RS, reduced endotoxemia, vascular TLR4 expression, and improved endothelial dysfunction.

Renal function plays a crucial role in the long-term control of BP, impaired activity in the kidney is undoubtedly involved in the prevalence of hypertension in SLE patients and murine models. Moreover, in this experiment, both fiber treatments decreased renal damage and BP concomitantly. Nonetheless, SLE-linked hypertension has been detected without displaying nephritis.^65,66^ Shaharir *et al*.^67^ showed that 53% of SLE patients in one cohort suffered from hypertension but not nephritis. Remarkably, fecal transplant from SLE induced an increase in BP that was not accompanied by changes in protein excretion, pointing to a kidney-independent BP regulatory role for the microbiota.

In conclusion, our study demonstrated that fiber interventions partially prevented the development of hypertension and ameliorated cardiac hypertrophy and kidney damage in a genetic model of SLE. These effects were associated with changes in the gut microbiota (increasing SCFAs-producing bacteria), improvement of gut integrity, and decreased endothelial dysfunction. Additionally, fecal inoculation from SLE mice treated with RS of ITF donor mice into recipients GF mice suppressed gut-immune system disbalance, endothelial dysfunction, and protects against hypertension. Overall, preventive BP effects and hypertensive cardiac damage induced by RS and ITF are partially attributed to improvement of the gut-immune system-vascular wall axis. Women get SLE approximately 9:1 over men. Due to this, our results for this experiment, and in most of the preceding bibliography were obtained using female models of the disease. Considering the evidence that supports significant differences in gut microbiota for both sexes,^68^ the possible effects of gut microbiota and its difference in BP regulation in males should be studied. Our results help rethink the current paradigm on the prevention of SLE-linked cardiovascular complications, suggesting modulation of the gut microbiota composition using fiber treatment. Nonetheless, caution is advisable for future extrapolations of our findings to humans since there are documented differences between the features of animal an human microbiota.

## Material and methods

### Animals and experimental groups

For animal protocols, we followed the National Institutes of Health Guide for the Care and Use of Laboratory Animals and got approval from the Ethics Committee of Laboratory Animals of the University of Granada (Spain) (Ref. 12/11/2017/164). In addition, our procedure conforms to the Guidelines for Transparency on Gut Microbiome Studies in Essential and Experimental Hypertension,^69^ and the ARRIVE guidelines.^70^ Considered that estrogens are relevant elements in both SLE disease and the associated hypertension in human and murine models,^54^ we utilized exclusively female mice.

*Experiment 1*: NZW/LacJ female mice (CTR group, *n* = 10) and NZBWF1 (*n* = 30) 25 weeks old, provided by Jackson Laboratories (RRID:SCR_004633, Bar Harbor, ME, USA), were used in this experiment. NZW/LacJ mice were used as control. NZBWF1 lupus mice were randomly assigned to three groups: SLE (no treatment group, *n* = 10), RS (SLE mice treated with SF11–025 diet: 72.7% insoluble fiber, from Specialty Feeds, Perth, Australia,^27^ *n* = 10) and ITF (SLE mice treated with ORAFTI P95, soluble fiber from Tener, Belgium,^57^ *n* = 10). The insoluble fiber was administered in the form of conventional pellets. The soluble fiber was diluted in the drinking water at a final dose of 250 mg/mouse/day. When the first stages of kidney dysfunction were observed by high proteinuria (at 25 week old) without high blood pressure, treatment with the fiber was initiated and continued then for 8 weeks.

In this experiment, all animals were housed in specific pathogen-free (SPF) facilities at University of Granada Biological Services Unit under standard laboratory conditions (12-h light/dark cycle, temperature 21–22°C, 50–70% humidity) in separate Makrolom cages (Ehret, Emmerdingen, Germany) to avoid horizontal transmission of bacteria, with dust-free laboratory bedding and enrichment. CTR, SLE, and ITF mice were provided with standard laboratory diet (SAFE A04, Augy, France) *ad libitum*. Water was changed every day, and both water and food intakes were analyzed daily. Studies were designed to generate equally sized groups and sufficient statistical power. Animals were randomly allotted to the four experimental groups and the experimenter was blinded to fiber treatment until data analysis was performed.

*Experiment 2*: To explore the involvement of microbiota in BP regulation, fecal inoculation to normotensive ten-week-old female C57Bl/6J germ-free (GF) mice (University of Granada, Granada, Spain) was performed.^17^ For this, we collected and pooled fresh stool samples from individual mice from all groups in experiment 1. The samples were used to generate a bacterial suspension by vigorous vortexing 1:20 in sterile phosphate-buffered saline (PBS) and centrifuged at 60 g for 5 min to eliminate the detritus. The suspension was aliquoted and stored at −80°C. Animals were randomly distributed among four different groups: GF with CTR microbiota (GF-C) (*n* = 8), GF with SLE microbiota (GF-SLE) (*n* = 10), GF with RS microbiota (GF-RS) (*n* = 10), and GF with ITF microbiota (GF-ITF) (*n* = 10). Inoculation was carried out consecutively twice in the first week. Then, mice were kept for 3 weeks. All GF mice were kept under sterile conditions at a gnotobiotic facility and were provided with standard laboratory diet *ad libitum*.

### Blood pressure measurements, physical characteristics, heart and kidney weight indices, and renal injury

SBP measurements were obtained from conscious, pre-warmed for 10–15 min at 35°C, restrained mice by tail-cuff plethysmography (Digital Pressure Meter, LE 5001; Letica S.A., Barcelona, Spain). Training and number of replicates were performed as described previously.^5^ At the end of the experiment 2, mice were subjected to isoflurane anesthesia, a polyethylene catheter containing 100 U heparin in isotonic, sterile NaCl solution was inserted in the left carotid artery to monitor intra-arterial BP. Twenty-four hours after the implantation of the catheter, we recorded intra-arterial BP uninterruptedly for 60 min with a sampling frequency of 400/s (McLab; AD Instruments, Hastings, United Kingdom). For intergroup comparisons, BP values recorded during the last 30 min were averaged.

We recorded body weights (in grams) for all groups. The left ventricle, liver, spleen, and kidney weight indices were calculated by dividing their weights by the tibia length. Samples were snap-frozen in liquid nitrogen and then stored at −80°C.

For histological studies, decapsulated kidneys from all groups were buffered, 10% formaldehyde-fixed, paraffin-embedded, and transversal sections in horizontal plane were stained with hematoxylin-eosin, Masson’s trichrome and periodic acid-Schiff stain. Histochemical stainings were interpreted and scored simultaneously by two independent investigators (F.O., and N.M-M.). The histological analysis was performed in blinded fashion on 4-micrometer sections with light microscopy, using the most appropriate stain for each lesion. Pathological changes in the kidney were assessed by evaluating glomerular activity (glomerular, endocapillary and extracapillary proliferation, karyorrhexis/fibrinoid necrosis, hyaline thrombi, cellular crescents, floccular synechia, wire loops, hyaline deposits, and fucsinophils deposits), tubulointerstitial activity (TLS interstitial inflammation, tubular cell necrosis, tubular casts, and flattening and tubular distension), and the chronicity of the lesions (fibrous crescents, glomerular sclerosis, tubular atrophy, and interstitial fibrosis), as previously reported.^71^ Sections were scored using a 0–3 scale for glomerular activity, as follows: 0 = no lesions, 1 = lesions in, 25% of glomeruli, 2 = lesions in 25–50% of glomeruli, and 3 = lesions in > 50% of glomeruli. Tubulointerstitial activity and lesion chronicity indices were scored using a 0–4 scale, as follows: 0 = no lesions, 1 = lesions in 1–10%, 2 = 11–25%, 3 = >25–50% and 4 = >50–100%. In the evaluation of mesangial sclerosis, <50% affected glomeruli were considered focal; diffuse> or equal to 50%; Segmental part of the glomerulus, and global > 50% of the glomerulus. Finally, was assessed the number of nuclei per glomerular cross-section (50 glomeruli without sclerosis per mouse). The mean scores for individual pathological features were summed to obtain the three main scores: the glomerular activity score, the tubulointerstitial activity score, and the chronic lesion score.

### Plasma, urine, and fecal parameters

We proceeded to the sacrifice of all animals at their respective experimental endpoints under isoflurane anesthesia. Plasma was obtained from blood samples and anti-ds-DNA antibody levels were measured in the aliquots as reported previously using an Alpha Diagnostic ELISA Kit (Alpha Diagnostic International, San Antonio, Texas, USA) according to the manufacturer’s instructions, as previously described.^21^ Additionally, we also analyzed LPS contents in plasma with the Pierce^TM^ chromogenic endotoxin quant kit (Thermo Fisher Scientific, Illinois, USA), following the manufacturer’s instructions. From spot urine, we determined proteinuria using the Combur Test strips (Roche Diagnostics, Mannheim, Germany).

Analysis of SCFA in mouse plasma and lyophilized stool, colon, and liver samples was performed by liquid chromatography-triple quadrupole-mass spectrometry (LC-QqQ-MS) with stable-isotope internal standard calibration after chemical derivatization with dansylhydrazine following an optimized and validated method based on Zhao *et al*.^72^ Analysis was carried out at CEMBIO, (Centre for Metabolomics and Bioanalysis, Madrid, Spain), with the LC Instrument 1260 Infinity series (Agilent Technologies), coupled to a Triple Quadrupole analyzer (G6470A, Agilent) with an electrospray ionization source in positive mode. Data were collected in dynamic multiple reaction monitoring.

### Vascular reactivity studies

Segments from aorta were loaded into a wire myograph (model 610 M, Danish Myo Technology, Aarhus, Denmark) with Krebs solution and in standard conditions for isometric tension measurement as described in detail in previous publications from our group.^73^ Precontraction was achieved with the thromboxane A_2_ analogous U46619 (3 nM). Serial relaxation curves were performed between washes in the absence and the presence of the specific pan-NOX inhibitor VAS2870 (10 µM), or the Rho kinase inhibitor Y27632 (0.5 µM) during 30 min. Results were expressed as relaxation levels in proportion to the precontraction tone.

### NADPH oxidase activity

NADPH oxidase activity was determined in vascular tissue with a lucigenin-enhanced chemiluminescence assay in intact aortic segments as previously described.^60^ Aortic segments from all groups were incubated for 30 min at 37°C in HEPES-based solution, adding NADPH (100 μM) to stimulate enzymatic activity. Measurements were recorded with a scintillation chamber (Lumat LB 9507, Berthold, Germany) in the presence of lucigenin (5 μM). NADPH oxidase activity is expressed as relative luminescence units (RLU)/min/mg dry aortic tissue.

### Reverse transcriptase-polymerase chain reaction (RT-PCR) and western blot analysis

RNA samples were obtained from colon, MLNs and aorta by homogenization and retrotranscribed into cDNA by standard methods to carry out RT-PCR determinations. Tissues were dissociated in 1 mL of PRImeZOL Reagent (Canvax Biotech, S.L., Córdoba, Spain) as we described previously.^60^ PCRs were performed using a PCRMax Eco 48 thermal cycler (PCRMax, Stone, Staffordshire, UK). A quantitative real-time RT-PCR technique was used to analyze mRNA expression. The forward and reverse probes employed are listed in Table 1. RT-PCRs were performed according to our established protocol using glyceraldehyde-3-phosphate dehydrogenase (GAPDH) as housekeeping. The analyses were performed through the ∆∆Ct method.^60^

**Table 1.** Oligonucleotides for real-time RT-PCR.

mRNA targets	Descriptions	Sense	Antisense
*MCT-1*	Monocarboxylate transporter 1	GTGCAGCAGCCAAGGAGCCC	CCATGGCCAGTCCGTTGGCC
*MCT-4*	Monocarboxylate transporter 4	CAGCTTTGCCATGTTCTTCA	AGCCATGAGCACCTCAAACT
*Hif-1a*	Hypoxia Inducible Factor 1 Subunit Alpha	ACCTTCATCGGAAACTCCAAAG	CTGTTAGGCTGGGAAAAGTTAGG
*TLR-4*	Toll-Like Receptor-4	GCCTTTCAGGGAATTAAGCTCC	AGATCAACCGATGGACGTGTAA
*GPR41*	G-protein-coupled receptor-41	CTTCTTTCTTGGCAATTACTGGC	CCGAAATGGTCAGGTTTAGCAA
*GPR43*	G-protein-coupled receptor-43	CGTTGGGGCTCAGAGGCGAC	TGCTCGGGAAGATCCGGGGG
*HDAC-3*	Histone Deacetylase 3	GCCAAGACCGTGGCGTATT	GTCCAGCTCCATAGTGGAAGT
*Occludin*	Occludin	ACGGACCCTGACCACTATGA	TCAGCAGCAGCCATGTACTC
*ZO-1*	Zonula occludens-1	GGGGCCTACACTGATCAAGA	TGGAGATGAGGCTTCTGCTT
*MUC-2*	Mucin-2	GATAGGTGGCAGACAGGAGA	GCTGACGAGTGGTTGGTGAATG
*MUC-3*	Mucin-3	CGTGGTCAACTGCGAGAATGG	CGGCTCTATCTCTACGCTCTCC
*IL-1β*	Interleukin 1 Beta	GCTACCTGTGTCTTTCCCGT	CATCTCGGAGCCTGTAGTGC
*TNF-α*	Tumor Necrosis Factor-alpha	CTACTCCCAGGTTCTCTTCAA	GCAGAGAGGAGGTTGACTTTC
*CX3CR1*	CX3C chemokine receptor 1	GAGTATGACGATTCTGCTGAGG	CAGACCGAACGTGAAGACGAG
*CD80*	CD80	TTCCCAGCAATGACAGACAG	CCATGTCCAAGGCTCATTCT
*CD86*	CD86	TCAATGGGACTGCATATCTGCC	GCCAAAATACTACCAGCTCACT
*Itga4*	Integrin alpha-4	TGTGCAAATGTACACTCTCTTCCA	CTCCCTCAAGATGATAAGTTGTTCAA
*Itgb7*	Integrin beta-7	AAACGGTGCTGCCCTTTGTAA	CTCTCTCTCGAAGGCTTGAGC
*IL-6*	Interleukin 6	CTCTGGGAAATCGTGGAAAT	TGTACTCCAGGTAGCTATGG
*HO-1*	Hemo-oxigenase-1	CCTCACTGGCAGGAAATCATC	CCTCGTGGAGACGCTTTACATA
*NQO-1*	NAD(P)H Quinone Dehydrogenase 1	TTCTCTGGCCGATTCAGAGT	GGCTGCTTGGAGCAAAATAG
*IL-1β*	Interleukin-1 beta	GCTACCTGTGTCTTTCCCGT	CATCTCGGAGCCTGTAGTGC
*IL-6 R*	Interleukin 6 Receptor	GCCACCGTTACCCTGATTTG	TCCTGTGGTAGTCCATTCTCTG
*RPL13a*	Ribosomal protein L13a	CCTGCTGCTCTCAAGGTTGTT	TGGTTGTCACTGCCTGGTACTT

We examined the content of ZO-1 and occludin in colonic homogenates by western blot analysis. These samples were run on a sodium dodecyl sulfate-polyacrylamide electrophoresis (20 μg of protein per lane). Next, the transference of proteins was performed to polyvinylidene difluoride membranes. The membranes were then incubated with the respective primary antibodies: rabbit polyclonal anti-occludin (Abcam, Cambridge, UK) and rabbit polyclonal anti-tight junction protein ZO-1 (Novus biological, Cambridge, UK). All were incubated at 1/1000 dilution overnight at 4°C. The membranes were then incubated with secondary peroxidase-conjugated goat antirabbit (1/10000; Santa Cruz Biotechnology). Antibody binding was detected by an ECL system (Amersham Pharmacia Biotech, Amersham, UK) and densitometric analysis was done by ImageJ software (version 1.52a, NIH, http://rsb.info.nih/ij/). Samples were re-probed for smooth muscle β-actin.

### Flow cytometry

MLNs, spleens, blood, and aorta were excised, homogenized and filtered to eliminate tissular debris. Erythrocytes were lysed with Gey’s solution. A protein transport inhibitor (BD GolgiPlug^TM^) was used according to the manufacturer’s instruction for an optimum detection of intracellular cytokines by flow cytometry, together with 50 ng/mL phorbol 12-myristate 13-acetate and 1 μg/mL ionomycin. Then, cells were blocked with anti-Fc-γ receptor antibodies to avoid masking by nonspecific binding (Miltenyi Biotec). The cells were stained for B cells (CD45+, B220+), Th17 (CD45+, CD3+ CD4+, IL-17A+), Th1 (CD45+, CD3+ CD4+, interferon (IFN)γ), and regulatory T cells (Tregs) (CD45+, CD3+, CD4+, CD25+). Key antibodies for flow cytometry are included in Table 2. Samples were analyzed through a flow cytometer Canto II (BD Biosciences) as previously described.^60,73^ Gate strategy for flow cytometry is shown in Figure S15.

**Table 2.** Key antibodies for flow cytometry.

Antibodies	Source
anti-CD45 (RRID:AB_2727597, FITC, clone 30-F11)	Miltenyi
anti-B220 (RRID:AB_398531, APC, clone RA3-6B2)	BD Bioscience
anti-CD3 (RRID:AB_2801803, PE, clone REA641)	Miltenyi
anti-CD4 (RRID:AB_1107001, PerCP-Cy5.5, clone RM4–5)	Invitrogen
anti-CD25 (RRID:AB_2784091, PE-VIO770, clone 7D4)	Miltenyi
anti-IL-17a (RRID:AB_1073235, PE-Cy7, clone eBio17B7)	eBioscience
anti-interferon-γ (IFN-γ; RRID:AB_2738165, PE-VIO770, clone XMG1.2)	eBioscience
viability dye (LIVE/DEAD® Fixable Aqua Dead Cell Stain)	Thermo Fisher

### Immunofluorescence

After the sacrifice, mouse colon tissues were isolated, fixed in 10% formalin overnight at 4°C, and paraffin-embedded. Paraffin cross-sections (5 μm) from fixed colons were prepared for immunofluorescence. Deparaffinized sections were rehydrated, boiled 3 min to retrieve antigens in 10 mM citrate buffer containing 0.05% Tween-20, pH6, and blocked for 1 h with 10% goat serum, 10% horse serum, plus 4% BSA in PBS. Samples were incubated with the following antibodies for immunofluorescence: rabbit monoclonal anti-FoxP3 (Cell Signaling, Danvers, MA, USA), and rat monoclonal anti-ROR gamma (eBioscience, San Diego, CA, USA). Secondary antibodies were polyclonal Alexa-Fluor-647-conjugated goat anti-rabbit or polyclonal Alexa-Fluor-568-conjugated goat anti-rat (Molecular Probes, Carlsbad, CA, USA). Sections were mounted with DAPI in Citifluor AF4 mounting medium (Aname; Madrid, Spain). The whole colonic cross-section was screened before taking representative pictures (minimum of three). Images were acquired at 512 × 512 pixels, 8 bits, using a Confocal TCS Leica SP5 microscope (Leica Microsystems GmbH; Wetzlar, Germany) fitted with a 40× oil-immersion objective. All images were processed for presentation with Photoshop (Adobe) and analyzed with ImageJ software.

### DNA extraction, 16S rRNA gene amplification, bioinformatics

DNA extraction was performed as previously documented from stool samples.^74^ Amplification was carried out on these samples for the V3-V4 region of the 16S rRNA gene.^75^ The resulting amplicons were analyzed and quantified with a Bioanalyzer 2100 (Agilent). The samples were then sequenced on an Illumina MiSeq instrument with 2 × 300 paired end read sequencing at the Unidad de Genómica (Parque Científico de Madrid, Madrid, Spain).

To process raw sequences, the barcoded Illumina paired-end sequencing (BIPES) pipeline was performed using the BIPES protocols.^76,77^ We utilized UCHIME (implemented in USEARCH, version 6.1) to screen out and remove chimeras in the *de novo* mode (using-minchunk 20-xn 7-noskipgaps 2).^78^

Further analyses were carried out with 16S Metagenomics (Version: 1.0.1.0) from Illumina. The sequences were subsequently clustered to an operational taxonomic unit (OTU) with USEARCH default parameters (USERACH61). The threshold distance was set to 0.03. Consequently, when similarities between 16S rRNA sequences were 97%, the sequences were classified as the same OTU. QIIME2-based alignments of representative sequences were carried out with PyNAST, and the SILVA database was used as the template file. The Ribosome Database project (RDP) algorithm was used to classify the representative sequences into specific taxa with the default database.^79^ The Taxonomy Database (National Center for Biotechnology Information) was used for classification and nomenclature. Bacteria were classified based on SCFAs end-product, as previously described.^80^ Briefly, genera were classified into more than one group if they were defined as producers of different metabolites.

### Statistical analysis

Shannon diversity, Chao richness, and Pielou evenness and observed species indexes were calculated with the PAST4.02 Palaeontological Statistics (PAST 4 ×). Reads in each OTU were normalized to total reads in each sample. Only taxa with a percentage of reads > 0.001% were used for the analysis. Partial least square discriminant analysis (PLS-DA) was also used on these data to determine significant taxonomic differences in two experimental groups, and VIP (variable importance in projection) scores were displayed to rank the ability of different taxa to discriminate between different groups. All data were analyzed with GraphPad Prism 8. Results are expressed as means ± SEM of measurements. The evolution of tail SBP and the concentration-response curves to acetylcholine were analyzed by two-way repeated-measures analysis of variance (ANOVA) with the Bonferroni *post hoc* test. The remaining variables were tested on normal distribution using Shapiro-Wilk normality test and compared using one-way ANOVA and Tukey *post hoc* test in case of normal distribution, or Mann-Whitney test or Kruskal-Wallis with Dunn’s multiple comparison test in case of abnormal distribution. *P* < 0.05 was considered statistically significant.

## Supplementary Material

Supplemental Material

## Data Availability

The sequencing dataset from this study have been deposited in ZENODO (Doi: 10.5281/zenodo.7547433).
