# Supplementary material for: Targeting the gut microbiota with dietary fibers: a novel approach to prevent the development cardiovascular complications linked to systemic lupus erythematosus in a preclinical study
Source: Gut Microbes. 2023 Aug 24;15(2):2247053. doi: 10.1080/19490976.2023.2247053 (PMC10453983; doi:10.1080/19490976.2023.2247053)
Supplement: Supplemental Material [file KGMI_A_2247053_SM0055.docx]

**Title: “Targeting the gut microbiota with dietary fibers: a novel approach to prevent the development cardiovascular complications linked to systemic lupus erythematosus in a preclinical study”**

Javier Moleón^a,b,*^, Cristina González-Correa^a,b,*^, Iñaki Robles-Vera^c^, Sofía Miñano^a^, Néstor de la Visitación^d^, Antonio Manuel Barranco^a,b^, Natividad Martín-Morales^e^, Francisco O’Valle^e,f^, Laura Mayo-Martínez^g^, Antonia García^g^, Marta Toral^a,b,f^, Rosario Jiménez^a,b,f^, Miguel Romero^a,b,†^, Juan Duarte^a,b,f,†^

Figure S1. Effects of fiber treatments on general parameters of systemic lupus erythematosus (SLE) mice. (A) Time course of body weight (n=9-10, data are shown as means ± SEM, *P<0.05 compared to the CTR group, two-way ANOVA, Sidak’s multiple comparisons test) (B) Mean of dairy drink, food, and energy intake in all experimental groups: control (CTR), SLE, and SLE-groups treated with resistant starch (RS) or inulin-type fructans (ITF). Values are expressed as means ± SEM at different timepoint.

Figure S2. Effects of fiber treatments in alpha diversity parameters and phyla composition of gut microbiota in systemic lupus erythematosus (SLE) mice. (A) Ecological parameters (B), proportion of bacterial phyla, (C) ratio of bacteria belonging to Firmicutes/Bacteroidetes (F/B) phyla, and (D) proportion of strict anaerobes bacteria in control (CTR), SLE, and SLE-groups treated with resistant starch (RS) or inulin-type fructans (ITF). Values are expressed as means ± SEM, n = 8-10, ^#^P<0.05 and ^##^P<0.01 compared to the untreated SLE group, one-way ANOVA.

Figure S3. Effects of fiber treatments in beta diversity parameters of gut microbiota in systemic lupus erythematosus (SLE) mice. (A) Two- and three-dimensional partial least square discriminant analysis (PLS-DA) of the bacterial community, which measures microorganism diversity between samples, at the level of the different taxa (phylum, class, order, family, genus, and species) (n = 8-10 mice per treatment group for each comparison) (B) VIP scores were used to rank the ability of different taxa to discriminate between two groups. A taxon with variable importance (VIP) score > 1 was considered important in the discrimination (n = 8-10 mice per group). Groups: control (CTR), SLE, and SLE-groups treated with resistant starch (RS) or inulin-type fructans (ITF).

Figure S4. Family changes in the gut microbiota composition induced by fiber treatments in systemic lupus erythematosus (SLE) mice. (A) Heat map of bacterial families. The heatmap colours represent the relative percentage of microbial genera assigned within each sample. (B) Relative abundance of bacterial families with a relative abundance > 1% in control (CTR), SLE, and SLE-groups treated with resistant starch (RS) or inulin-type fructans (ITF). Values are expressed as means ± SEM, n = 8-10, *P<0.05 and **P<0.01 compared to the CTR group, ^#^P<0.05 and ^##^P<0.01 compared to the untreated SLE group, one-way ANOVA.

Figure S5. Genera changes in the gut microbiota composition induced by fiber treatments in systemic lupus erythematosus (SLE) mice. (A) Heat map of bacterial genus. The heatmap colours represent the relative percentage of microbial genera assigned within each sample. (B) Relative abundance of bacterial genus with a relative abundance > 1% in control (CTR), SLE, and SLE-groups treated with resistant starch (RS) or inulin-type fructans (ITF). Values are expressed as means ± SEM, n = 8-10, **P<0.01 compared to the CTR group, ^#^P<0.05 and ^##^P<0.01 compared to the untreated SLE group, one-way ANOVA.

Figure S6. Changes in significant species in the gut microbiota composition induced by fiber treatments in systemic lupus erythematosus (SLE) mice. Groups: control (CTR), SLE, and SLE-groups treated with resistant starch (RS) or inulin-type fructans (ITF). Values are expressed as means ± SEM, n = 8-10, ^##^P<0.01 compared to the untreated SLE group, one-way ANOVA.

Figure S7. Changes in the liver concentration of SCFAs induced by fiber treatments in systemic lupus erythematosus (SLE) mice measured by HPLC-ESI-MS and expressed as µmol/g of lyophilized liver. Groups: control (CTR), SLE, and SLE-groups treated with resistant starch (RS) or inulin-type fructans (ITF). Values are expressed as means ± SEM, n = 7-10, ^##^P<0.01 compared to the untreated SLE group, one-way ANOVA.

Figure S8. Fiber treatments improved colonic epithelial integrity markers, permeability, and inflammation in systemic lupus erythematosus (SLE) mice. (A) Colonic mRNA levels and protein expression of barrier-forming proteins occludin and zonula occludens-1 (ZO-1), and (B) mucins (MUC)-2 and MUC-3. (C) Plasma LPS levels measured as endotoxin units (EU). (D) Colonic mRNA expression levels of proinflammatory cytokines interleukin (IL)-1ß and tumour necrosis factor (TNF)α. (E) Colonic mRNA levels of G-protein coupled receptors (GPR)43, and histone deacetylases (HDAC)3. Groups: control (CTR), SLE, and SLE-groups treated with resistant starch (RS) or inulin-type fructans (ITF). Values are expressed as means ± SEM, n = 9-10, *P<0.05 and **P<0.01 compared to the CTR group, ^#^P<0.05 and ^##^P<0.01 compared to the untreated SLE group, one-way ANOVA.

****Figure S9. Fiber treatments prevented T cells activation in mesenteric lymph nodes in systemic lupus erythematosus (SLE) mice. (A) mRNA levels of the marker of antigen presenting cells CX3CR1+. (B) Expression of the B7 ligands CD80 and CD86, and (C) T lymphocytes integrinα4β7 (Itga4, Itgb7). (D) mRNA levels of interleukin (IL)6. (E) Expression of G protein-coupled receptor (GPR)43, and (F) histone deacetylase (HDAC)3. (G) Pathway nuclear factor erythroid 2-related factor 2 (Nrf2)/heme oxygenase 1 (HO-1)/IL-6 receptor measured by the mRNA levels of HO-1, NAD(P)H:quinone oxidoreductase 1 (NQO1), and IL-6 receptor (IL-6R). Groups: control (CTR), SLE and SLE-groups treated with resistant starch (RS) or inulin-type fructans (ITF). Values are expressed as means ± SEM, n = 9-10, *P<0.05 and **P<0.01 compared to the CTR group, ^#^P<0.05 and ^##^P<0.01 compared to the untreated SLE group, one-way ANOVA.

Figure S10. Fiber treatments prevented T cells polarization in lamina propria in systemic lupus erythematosus (SLE) mice. A) Representative images of ROR**γ** immunofluorescence (yellow), FoxP3 (magenta) and DAPI-stained nuclei (cyan). Scale bar 100 µm (top), and 50 µm (bottom). B) Quantification of ROR**γ** and Foxp3 immunofluorescence in colonic sections from all experimental groups. Groups: control (CTR), SLE and SLE-groups treated with resistant starch (RS) or inulin-type fructans (ITF). Values are expressed as means ± SEM, n = 3, *P<0.05 compared to the CTR group, ^#^P<0.05 compared to the untreated SLE group, one-way ANOVA.

Figure S11. Effects of fiber treatments on lymphocytes populations in systemic lupus erythematosus (SLE) mice. (A) Regulatory T cells (Treg), Th17, and Th1 cells measured by flow cytometry in spleen, and (B) B cells, Tregs, Th17, and Th1 cells measured by flow cytometry in blood from control (CTR), SLE and SLE-groups treated with resistant starch (RS) or inulin-type fructans (ITF). All data are expressed as % of parent, except for B cells, that are represented as % of grandparent (% of CD45+). Values are expressed as means ± SEM, n = 9-10, *P<0.05 and **P<0.01 compared to the CTR group, ^#^P<0.05 compared to the untreated SLE group, one-way ANOVA.

Figure S12. Fiber treatments prevented the transfer of altered gut permeability phenotype to germ-free mice induced by inoculation of faeces from systemic lupus erythematosus (SLE) mice. (A) Colonic mRNA expression levels of barrier-forming proteins occludin and zonula occludens-1 (ZO-1), and (B) mucins (MUC)-2 and MUC-3. (C) Plasma LPS levels measured as endotoxin units (EU). (D) Colonic mRNA expression levels of proinflammatory cytokines interleukin (IL)-1ß and tumour necrosis factor (TNF)α. (E) Colonic mRNA levels of G-protein coupled receptors (GPR)43, and histone deacetylases (HDAC)3. Groups: germ-free (GF) inoculated with control faeces (GF-CTR), GF inoculated with SLE faeces (GF-SLE) and GF inoculated with faeces from SLE-groups treated with resistant starch (GF-RS) or with inulin-type fructans (GF-ITF). Values are expressed as means ± SEM, n = 8-10, *P<0.05 compared to the GF-CTR group, ^#^P<0.05 and ^##^P<0.01 compared to the GS-SLE group, one-way ANOVA.

Figure S13. Fiber treatments prevented the transfer T cells activation in mesenteric lymph nodes phenotype to germ-free mice induced by inoculation of faeces from systemic lupus erythematosus (SLE) mice. (A) mRNA levels of the marker of antigen presenting cells CX3CR1+, the B7 ligands CD80 and CD86, the T lymphocytes integrinα4β7 (Itga4, Itgb7), and the interleukin (IL)6. (B) Expression of G protein-coupled receptor (GPR)43, and (F) histone deacetylase (HDAC)3. (C) Pathway nuclear factor erythroid 2-related factor 2 (Nrf2)/heme oxygenase 1 (HO-1)/IL-6 receptor measured by the mRNA levels of HO-1, NAD(P)H:quinone oxidoreductase 1 (NQO1), and IL-6 receptor (IL-6R). Groups: germ-free (GF) inoculated with control faeces (GF-CTR), GF inoculated with SLE faeces (GF-SLE) and GF inoculated with faeces from SLE-groups treated with resistant starch (GF-RS) or with inulin-type fructans (GF-ITF). Values are expressed as means ± SEM, n = 8-10, *P<0.05 compared to the GF-CTR group, ^#^P<0.05 and ^##^P<0.01 compared to the GS-SLE group, one-way ANOVA.

Figure S14. Fiber treatments prevented the transfer of Th17 differentiation phenotype to germ-free mice induced by inoculation of faeces from systemic lupus erythematosus (SLE) mice. Proportion of different immune cell types (Total B lymphocytes, Regulatory T cells (Tregs), Th17, and Th1 cells measured by flow cytometry in (A) mesenteric lymph nodes, (B) spleen, and (C) blood. All data are expressed as % of parent, except for B cells, that are represented as % of grandparent (% of CD45+). Groups: germ-free (GF) inoculated with control faeces (GF-CTR), GF inoculated with SLE faeces (GF-SLE) and GF inoculated with faeces from SLE-groups treated with resistant starch (GF-RS) or with inulin-type fructans (GF-ITF). Values are expressed as means ± SEM, n = 8-10, *P<0.05 and **P<0.01 compared to the GF-CTR group, ^#^P<0.05 and ^##^P<0.01 compared to the GS-SLE group, one-way ANOVA.

Figure S15. Gating strategy for flow cytometry.
